# Leri–Weill Dyschondrosteosis Caused by a Leaky Homozygous *SHOX* Splice-Site Variant

**DOI:** 10.3390/genes14040877

**Published:** 2023-04-07

**Authors:** Julia Vodopiutz, Lisa-Maria Steurer, Florentina Haufler, Franco Laccone, Dorota Garczarczyk-Asim, Matthias Hilkenmeier, Philipp Steinbauer, Andreas R. Janecke

**Affiliations:** 1 Department of Pediatrics and Adolescent Medicine, Division of Pediatric Pulmonology, Allergology and Endocrinology, Comprehensive Center for Pediatrics, Medical University of Vienna, 1090 Vienna, Austria; 2 Vienna Bone and Growth Center, 1130 Vienna, Austria; 3 Department of Pediatrics and Adolescent Medicine, Division of Neonatology, Pediatric Intensive Care and Neuropediatrics, Comprehensive Center for Pediatrics, Medical University of Vienna, 1090 Vienna, Austria; 4 Institute of Medical Genetics, Medical University of Vienna, 1090 Vienna, Austria; 5 Department of Pediatrics I, Medical University of Innsbruck, 6020 Innsbruck, Austria; 6 Division of Human Genetics, Medical University of Innsbruck, 6020 Innsbruck, Austria

**Keywords:** SHOX, skeletal disorder, short stature, leaky splice-site, precision medicine, gene dosage, haploinsufficiency, pseudo-autosomal inheritance

## Abstract

SHOX deficiency is a common genetic cause of short stature of variable degree. SHOX haploinsufficiency causes Leri–Weill dyschondrosteosis (LWD) as well as nonspecific short stature. *SHOX* haploinsufficiency is known to result from heterozygous loss-of-function variants with pseudo-autosomal dominant inheritance, while biallelic *SHOX* loss-of-function variants cause the more severe skeletal dysplasia, Langer mesomelic dyschondrosteosis (LMD). Here we report for the first time the pseudo-autosomal recessive inheritance of LWD in two siblings caused by a novel homozygous non-canonical, leaky splice-site variant in intron 3 of *SHOX:* c.544+5G>C. Transcript analyses in patient-derived fibroblasts showed homozygous patients to produce approximately equal amounts of normally spliced mRNA and mRNA with the abnormal retention of intron 3 and containing a premature stop codon (p.Val183Glyfs*31). The aberrant transcript was shown to undergo nonsense-mediated mRNA decay, and thus resulting in SHOX haploinsufficiency in the homozygous patient. Six healthy relatives who are of normal height are heterozygous for this variant and fibroblasts from a heterozygote for the c.544+5G>C variant produced wild-type transcript amounts comparable to healthy control. The unique situation reported here highlights the fact that the dosage of SHOX determines the clinical phenotype rather than the Mendelian inheritance pattern of *SHOX* variants. This study extends the molecular and inheritance spectrum of SHOX deficiency disorder and highlights the importance of functional testing of *SHOX* variants of unknown significance in order to allow appropriate counseling and precision medicine for each family individual.

## 1. Introduction

The short-stature-homeobox gene *SHOX* has been recognized as a major human growth gene [1], encoding for a nuclear transcription factor crucial in regulating chondrogenesis in human growth plates [2]. Genetic variants of *SHOX* causing dosage alterations in SHOX expression result in a broad spectrum of human phenotypes with abnormal growth and skeletal development, with a cumulative prevalence of at least 1:1000 [3]. Alterations of SHOX dosage are thought to contribute to the growth phenotypes in disorders caused by aberrant numbers of sex chromosomes such as Turner and Klinefelter syndrome, respectively [1,3,4,5].

Heterozygous *SHOX* variants leading to haploinsufficiency result in non-syndromic SHOX-deficient short stature (SS, MIM 300582) at the mild end and Leri–Weill dyschondrosteosis (LWD, MIM 127300) at the severe end of the clinical spectrum [3,6]. SS is defined as short stature without skeletal anomalies, while LWD is characterized by the clinical triad of short stature, Madelung deformity of the wrist, and mesomelia. Madelung deformity and mesomelia increase with age in severity and frequency and are typically not recognized before the age of 6 years. Scoliosis, increased BMI, hypertrophy of calf muscles, short fourth metacarpals, high-arched palate, or increased carrying angle of the elbow are variable features of LWD [3,6,7]. Short stature in SHOX haploinsufficiency is usually mild to moderate ranging from 3.08 to 2.94 SDS below the mean [3,8,9]. Growth hormone therapy (GHT) was shown to be an effective treatment option for short stature in prepubertal individuals with SHOX haploinsufficiency [10,11]. In contrast to SHOX haploinsufficiency, total loss of SHOX due to biallelic *SHOX* null variants is rare and causes the severe skeletal disorder, Langer mesomelic dysplasia (LMD; MIM 249700) [12]. LMD is characterized by short stature with final heights between 5.5 and 8.9 SDS below the mean, severe defects of long tubular bones with marked rhizo-mesomelic shortening of extremities, and aplasia or severe hypoplasia of the ulna and fibula and thickened radius and tibia. Madelung deformity is not a constant feature of LMD [3,12,13]. Cognitive development is unaffected in all types of SHOX deficiency [3].

SHOX deficiency disorders are transmitted in a pseudo-autosomal pattern and are not dependent on X inactivation, as *SHOX* is located within the telomeric pseudo-autosomal region 1 (PAR1) of the shorter arm of both sex chromosomes. So far, pseudo-autosomal dominant inheritance was ascribed to LWD and SS, respectively [3,4], while the LMD phenotype was shown to result from biallelic LWD causing *SHOX* variants transmitted form two heterozygotes with a LWD phenotype [3,7,13].

Here we report for the first time pseudo-autosomal recessive inheritance of LWD in two siblings caused by a novel homozygous non-canonical, leaky splice-site variant in intron 3 of *SHOX,* ultimately resulting in SHOX haploinsufficiency, as demonstrated on the transcript level. The unique situation reported here highlights that the dosage of SHOX determines the clinical phenotype rather than the Mendelian inheritance pattern of *SHOX* variants.

## 2. Materials and Methods

### 2.1. Ethics and Patient Samples

Written informed consent for molecular genetic studies and publication of clinical data were obtained from all participants, and the ethics committees of the Medical Universities of Innsbruck approved the study. Genomic DNA was extracted from peripheral blood leukocytes from all participants by standard procedures and primary fibroblasts from patient P1, her healthy mother and from a healthy unrelated control were obtained by skin biopsy.

### 2.2. Clinical Assessment

All participants (P1, P2, and 13 relatives) were evaluated by a pediatrician-clinical geneticist (author’s initials: J.V.) with particular attention to clinical signs of SHOX deficiency. The evaluation included medical and family history, review of past medical records, and a physical examination with documentation of standing height (H), weight (W), sitting height (SH), arm span (AS), and head circumference (HC). Subischial leg length (LL, defined as difference between H and SH), Body mass index (BMI), and ratios of AS/H and SH/H were calculated for each individual. These body measurements and ratios were stratified according to sex and age-related reference standards and expressed as standard deviation scores (SDS). SDS of adults were calculated using the same references at 19 years of age. For H, W, SH, and HC Austrian references were applied [14]. As the SDS of AS and ratios are not available in the Austrian references, the following international references were applied for AS [15], SH/H [16], and AS/H [17,18]. Box blot analysis was performed for graphically demonstrating genotype-based growth measurements within this pedigree using Jamovi 2.3.21.0. In subjects P1 and P2, a complete skeletal radiograph investigation and blood endocrinology (IGF1, IGFBP3, GH) and chemistry (electrolytes, kidney function and liver function tests, glucose levels) analyses were performed.

### 2.3. Detection of SHOX Variants

Genomic DNA was extracted from whole blood samples using the QIA Symphony kit (Qiagen, Hilden, Germany) following the manufacturer’s instructions.

Diagnostic testing for deletions and duplications of *SHOX* exons and regulatory elements was performed in the index patient P1 using the commercial kit SALSA MLPA P018-G1 *SHOX* probemix (MRC-Holland) according to manufacturer’s instructions. This MLPA test contains 26 probes spaced 0.2–6.7 kb in the coding region and 0.4–338 kb in the non-coding region. The amplified fragments were analyzed by capillary electrophoresis on an ABI PRISM 3100 Genetic Analyzer and the data were analyzed with JSI software (Kippenheim, Germany).

*SHOX* sequence analysis was performed with the genomic DNA sample from P1 by direct sequencing of the coding exons (NM_000451.4 exons 1–5 https://www.ncbi.nlm.nih.gov/nuccore/NM_000451.4, NM_006883.2 exon 6 https://www.ncbi.nlm.nih.gov/nuccore/NM_006883.2). Each exon was PCR amplified using GoTaq G2 Flexi DNA Polymerase (Promega, Madison, WI, USA) with published PCR primers (Fanelli et al. 2020). PCR products were visualized on a 1.5% agarose gel, purified using EuroSap—PCR enzymatic clean-up kit (Euroclone, Milan, Italy), and then sequenced in the forward or reverse direction with the BigDye Terminator v1.1 Cycle Sequencing Kit (Applied Biosystems, Waltham, MA, USA) and analyzed on an ABI PRISM 3100 Genetic Analyzer (Applied Biosystems), and data were analyzed with JSI software (Kippenheim, Germany).

For the segregation analysis of an intron 3 splice-site variant detected in the proband, Sanger sequencing of exon 3 and flanking intronic sequences of *SHOX* was performed in samples from the affected sib P2, and from 13 healthy relatives. To exclude other monogenetic causes underlying the mild skeletal dysplasia in the index patient, whole exome, paired-end sequencing (WES) was performed in P1 after target enrichment in 1 µg of genomic DNA with the 36.8-Mb Twist Comprehensive Exome Panel (Twist Bioscience, San Francisco, CA, USA) using an Illumina Hi-Seq2000 platform. WES variants were identified with SeqNext (Version 5.0; JSI). WES variants were filtered for an allele frequency of <0.005 in the dbSNP (http://www.ncbi.nlm.nih.gov/projects/SNP/ (last accessed on 16 February 2023)), National Heart, Lung, and Blood Institute (http://evs.gs.washington.edu/EVS), Exome Aggregation Consortium (http://exac.broadinstitute.org/ (last accessed on 16 February 2023)), and gnomAD databases. The WES dataset was investigated for variants 1) by conducting a gene panel analysis using the Genomicsengland gene panel #309 (https://panelapp.genomicsengland.co.uk/panels/309/ (last accessed on 16 February 2023)) and by HPO analysis (Short stature (HP:0004322), skeletal dysplasia (HP:0002652)) using the VarSeak (JSI) software.

Missense variants were evaluated in silico for pathogenicity by PolyPhen-2 (http://genetics.bwh.harvard.edu/pph2 (last accessed on 16 February 2023)) and CADD (http://cadd.gs.washington.edu/score (last accessed on 16 February 2023)).

### 2.4. SHOX Transcript Analysis

Patient and control skin fibroblasts were cultured using standard protocols; total RNA was extracted using the Qiagen RNAeasy mini kit (Qiagen, Hilden) and cDNA was transcribed with superscript III (Invitrogen, Waltham, MA, USA). Protein synthesis inhibitor puromycin in a concentration of 20 mg/12 mL was added to patient and control fibroblast cultures 4 h before harvesting for total RNA extraction.

To address the putative functional consequences of a variant in the intron 3 donor splice-site, a semi-quantitative reverse transcriptase (RT)-PCR reaction was performed with a forward primer binding to NM_000451.4 exon 1 (https://www.ncbi.nlm.nih.gov/nuccore/NM_000451.4) (5′-GCATTTGTTCAAGGACCACG) and a reverse primer binding to exon 5 (5′-CTGTTGCTTTTGGCGGC). cDNA-derived PCR products were sequenced by conventional Sanger sequencing using Big Dye terminator chemistry on an ABI PRISM 3100 Genetic Analyzer (Applied Biosystems), and data were analyzed with JSI software (Kippenheim, Germany).

The relative amounts of aberrant SHOX transcript vs. wild-type transcript were assessed by qPCR using a QuantStudio3 system (Thermofisher, Waltham, MA, USA), the Maxima SYBR Green/ROX qPCR Master Mix (Thermofisher), the exon 2-exon 3 junction spanning forward primer EX2_3_f 5′-GCGTGCAGGTTTGGTTCC combined with wild-type allele-specific reverse primer EX3_4_r 5′-CCAAGATGACGCCTTTATGC, and with mutation-specific primer EX3_IN3_r 5′-CCGACAGCCACCTTTATGC, producing fragments of 78 bp and 77 bp, respectively. Amounts were normalized to housekeeping gene PPIB (PPIB_cDNA_f2 5′-AACATGAAGGTGCTCCTTGC and PPIB_cDNA_r2 5′-AGGTCAAAATACACCTTGACGG).

## 3. Results

### 3.1. Clinical Characteristics

#### 3.1.1. Patients 1 and 2

**Figure 1:**
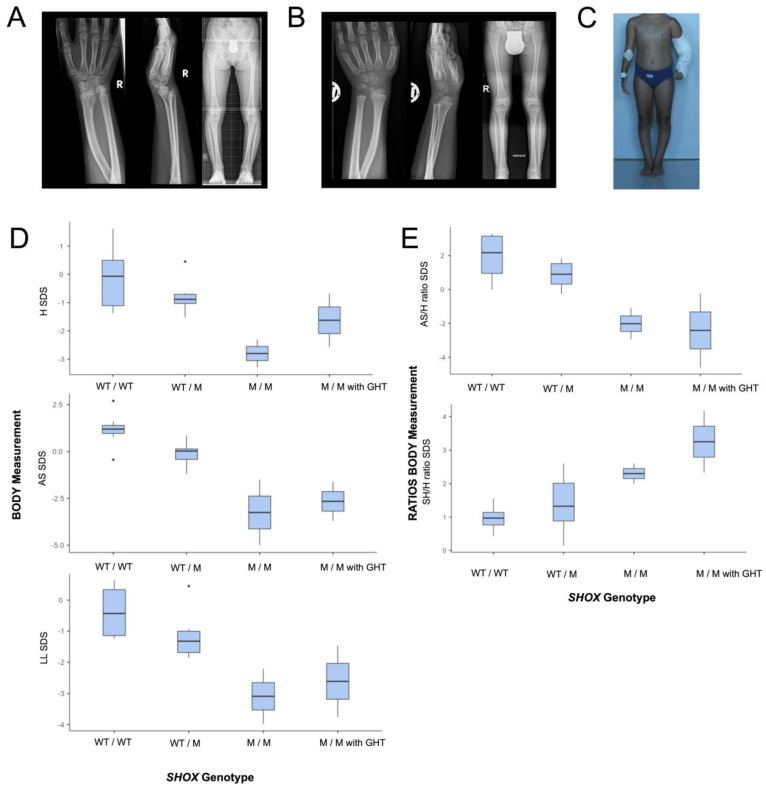
Clinical features in two siblings with biallelic SHOX variants. (**A**,**B**): Madelung deformity and mild mesomelia but absence of LMD characteristics on skeletal radiographs in P1 and P2. (**C**) LWD phenotype with Madelung deformity, mild mesomelic shortening of extremities, genu varum in P1 at age 12 years. (**D**,**E**): Genotype-based blotting of body measurements and ratios of 15 individuals from the pedigree show homozygotes having short stature with mesomelic shortening of the upper and lower extremities. Heterozygotes have normal H but predominantly cluster in the lower normal range for H and length of extremities when compared to wild-types. Genotype groups are defined as follows: wild-type (WT/WT) vs. heterozygotes (WT/M), vs. homozygotes without GHT (M/M), vs. homozygotes with GHT (M/M with GHT).

Two siblings (P1, P2) from healthy Austrian parents who deny any consanguinity were referred in infancy for evaluation of mild short stature and variable skeletal anomalies. Both siblings were finally diagnosed with an LWS phenotype based on clinical and radiological features. Mild short stature with mesomelic shortening of the upper and lower extremities and Madelung deformity were recognizable in both siblings from the age of 7 years and increased in severity with age. After establishing the diagnosis of SHOX deficiency, GHT was initiated in both individuals at the age of 12 years (P1) and 7.5 years (P2), respectively, and resulted in increased growth velocity and height (−3.3 SDS vs. −2.5 SDS after 3 years GHT in P1; −2.3 SDS vs. −0.68SDS after 4.5 years GHT in P2). Neither rhizomelia nor severe defects of long tubular bones, severe short stature, micrognathia, or facial dysmorphism were present in P1 and P2. (Figure 1).

P1 is the first child of healthy parents who was born at 38 weeks gestational age, after an uneventful pregnancy, with normal birth length (−1.3 SDS). At age 11.9 years, she presented with H 131.1 cm (−3.3 SDS), BMI 18.3 (0.13 SDS), SH 72.3 cm (−1.91 SDS), LL 58.8 cm (−3.97 SDS), AS 123 cm (−5 SDS), SH/H ratio 0.551 (2.6 SDS), SH/LL ratio 1.23 (2.38 SDS), and AS/H ratio 0.94 (−2.94 SDS). Bilateral Madelung deformity required orthopedic surgery at the age of 11.5 years. Genu varum was treated bilaterally by epiphysiodesis of the lateral tibia at the age of 13.5 years and by High Tibial Osteotomy at the age of 17.5 years. P1 received GHT from age 12 years to 15 years and she presented with final adult body measurements at the age of 17.2 years with H 151.3 cm (−2.57 SDS), BMI 20.4 (−0.33 SDS), HC 54.0 cm (−0.1 SDS), SH 87.7 cm (0.07 SDS), LL 63.6 cm (−3.76 SDS), AS 137.8 cm (−3.7 SDS), SH/LL ratio 1.379 (3.75 SDS), and SH/H ratio 0.58 (4.17 SDS) and AS/H ratio 0.91 (−4.6 SDS).

P2 is the younger brother of P1 and was born at 37 weeks gestational age with borderline birth length (−2.05 SDS). He was evaluated at the age of 6.8 years as part of the clinical family investigation when his sister P1 was first evaluated for short stature. At the age 7.4 years he presented with H 114.2 cm (−2.3 SDS), BMI 16.3 kg (0.49 SDS), HC 51.0 cm (0.32 SDS), SH 64.2 cm (−1.46 SDS), LL 51 cm (−2.21 SDS), AS 111.2 cm (−1.5 SDS), SH/LL ratio 1.259 (1.51 SDS), SH/H ratio 0.562 (2 SDS). An increased carrying angle of the elbow and subtle radiologic signs of Madelung deformity were noted on skeletal radiographs. Limited elbow mobility, Madelung deformity, and mesomelic disproportion of upper and lower limbs became more evident with puberty but did not require surgery. He started GHT at the age of 7.5 years and at age 11.9 years he presented with H 147.1 cm (−0.68 SDS), BMI 19.27 (0.76 SDS), HC 54.2 cm (−0.1 SDS), SH 79.8 cm (0.42 SDS), LL 67.3 cm (−1.46 SDS), AS 141 cm (−1.61 SDS), SH/LL ratio 1.186 (2.17 SDS), SH/H ratio 0.542 (2.33 SDS), and AS/H ratio 0.96 (−0.23 SDS).

#### 3.1.2. Clinical Investigations and Body Measurements in the Whole Pedigree

**Figure 2:**
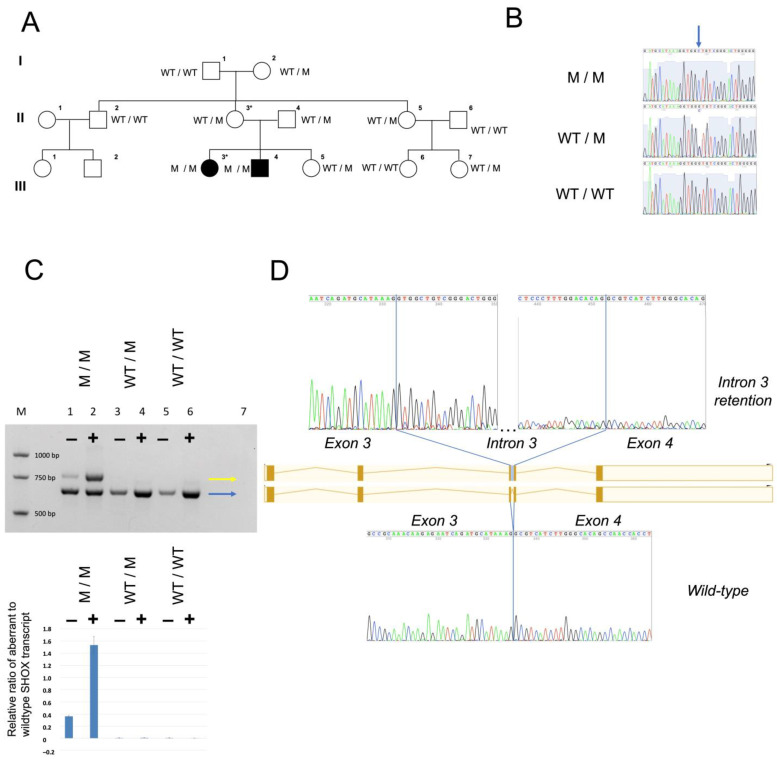
Pedigree, *SHOX* c.544+5G>C variant detection and consequences on the transcript level. (**A**) Simplified pedigree and segregation of the *SHOX* variant. (**B**) Sequence chromatograms to demonstrate the novel *SHOX* variant (arrow). (**C**) Genotype-based transcript analysis in cDNA shows that the homozygous *SHOX* c.544+5G>C variant leads to production of both wild-type and an aberrant transcript, which retains intron 3 ((**C**) upper panel). The abnormal transcript undergoes NMD as demonstrated by RT-PCRs in cDNA obtained from fibroblasts with (+) and without (−) NMD inhibition with puromycin. M/M, M/WT, WT/WT represent homozygous mutant, heterozygous mutant and wild-type genotypes, M denotes a molecular size marker, lane 7 No template control. In M/M, a ratio of aberrant SHOX transcript vs. wild-type transcript of 1.54 in (+) puromycin-treated patient cells (+), and of 0.36 in non-treated (-) cells was found, assessed by allele-specific qPCRs; the relative ratio of aberrant to wild-type SHOX transcript is shown (means and standard deviations of four technical replicates) ((**C**) lower panel)). Sanger sequencing reveals wild-type splicing of all *SHOX* exons of the RT-PCR product common to patient, parent and control (blue arrow) and reveals retention of intron 3 in the aberrant fragment seen in the patient only (yellow arrow) (**D**).

Thirteen relatives (female = 8, male = 5), of whom six were later identified to be carrier (female = 5, male = 1), were clinically investigated, with emphasis on mild SHOX deficiency features. (Figure 2A) All thirteen relatives were healthy, had no Madelung deformity, and were of a stature within the normal range. In both parents of P1 and P2, skeletal radiographs of both hands and forearms excluded also subtle signs of Madelung deformity. Genotype-based comparison of body measurements within the 15 individuals of the pedigree revealed both patients to be the only individuals with short stature and relatively shortened extremities. In the six heterozygotes H, AS and LL predominantly clustered in the lower normal range when compared to wild-types, while SH was similar between wild-type and heterozygotes. (Figure 1D,E)

### 3.2. Identification of a Novel Homozygous Splice-Site SHOX Variant

LWD was suggested by clinical phenotyping and therefore targeted sequencing analysis and MLPA-based deletion/duplication analysis of *SHOX* were performed. Sequencing identified homozygosity for the novel intronic *SHOX* splice-site variant c.544+5G>C in both LWS affected patients. This variant is neither listed in population database gnomAD nor in the ClinVar mutation database, and its CADD Phred score is 16.2. ACMG criteria PM2, PP1, PP3, PS3, PP1-S apply to c.544+5G>C, and result in a classification as likely pathogenic. The analysis using MRC-Holland’s SALSA MLPA probemix P018 excluded a deletion or duplication of SHOX exons and regulatory regions (Supplemental Figure S1). Familial segregation analysis identified six healthy family members with normal body height to be heterozygotes for this variant, in keeping with autosomal recessive inheritance of the LWS phenotype in this family (Figure 2A,B). As autosomal recessive inheritance of LWD is uncommon, chromosomal microarray analysis, conventional karyotyping, and WES were performed in P1 in order to widely exclude likely pathogenic and pathogenic variants in known skeletal dysplasia genes and relevant copy-number variants in the genome. WES identified homozygosity for the *SHOX* splice-site mutation c.544+5G>C as the only deleterious variant in genes related to skeletal disorders in P1 (Supplemental Results in Supplementary Materials).

### 3.3. The SHOX Variant Represents a Leaky Splice Donor Site Mutation

The *SHOX* c.544+5G>C variant identified in our study was predicted to weaken the donor site of intron 3 by in silico splice prediction algorithms. (Supplemental Figure S2) However, because this variant was not located within the canonical splice-site, we generated experimental evidence to support its potential pathogenicity. We examined the effects of the c.544+5G>C variant in cDNA obtained from skin fibroblast cultures from patient P1 and from one carrier, respectively.

The homozygous *SHOX* c.544+5G>C variant leads to production of approximately equal amounts of wild-type and an aberrant transcript which retains intron 3 and contains a premature stop codon (p.Val183Glyfs*31). The abnormal transcript undergoes nonsense-mediated mRNA decay (NMD) as demonstrated by RT-PCRs in cDNA obtained from fibroblasts with (+) and without (−) NMD inhibition with protein synthesis inhibitor puromycin prior to cell harvesting (Figure 2C). The ratio of aberrant SHOX transcript to wild-type transcript was assessed by allele-specific qPCRs, and a mean ratio of 1.54 in puromycin-treated patient cells and a ratio of 0.36 in non-treated cells were found. The abnormal transcript was not detected in cDNA from a heterozygous variant carrier, and transcript levels were comparable to the control (Figure 2C). We speculate that abnormal SHOX transcript is produced in fibroblasts heterozygous for the c.544+5G>C variant, but to a lesser amount than in homozygotes, and that NMD inhibitor treatment in our study was insufficient to detect minor amounts of aberrant transcript. Another possibility of not detecting the aberrant transcript in a heterozygous state could be a preferential amplification of the smaller transcript (wild-type). Collectively, our data suggest that the identified non-canonical, leaky splice-site mutation in intron 3 of *SHOX* causes haploinsufficiency in the homozygous state.

## 4. Discussion

We report here the unique situation of pseudo-autosomal recessive inheritance of LWD in a brother and sister with a novel intronic *SHOX* splice-site mutation c.544+5G>C. Transcript analysis demonstrated that this *SHOX* variant creates a leaky splice donor site, resulting in production of similar amounts of wild-type and aberrant, NMD-sensitive, transcripts. Thus, haploinsufficiency of the SHOX transcript results from a homozygous leaky *SHOX* variant and produces the LWD phenotype in homozygotes for this variant, with regard to the extent of short stature and skeletal involvement. However, not having the SHOX protein levels as well as no aberrant transcript detection in the NMD inhibitor treated condition in heterozygous state are limitations of this study. 

Six healthy relatives were shown to be heterozygous for the *SHOX* c.544+5G>C variant and fibroblasts of a heterozygote were shown to produce an amount of wild-type transcript comparable to healthy control. Accordingly, none of the heterozygotes fulfill the criteria of LWD or SS, supporting the idea that the leaky *SHOX* splice-site c.544+5G>C variant is sufficient to prevent heterozygotes from SHOX haploinsufficiency. Nevertheless H, AS, and LL of heterozygotes predominantly cluster in the lower normal range when compared to wild-type family members. While leaky splice-site variants have been proven to contribute to phenotypic variation in other monogenetic human diseases [19,20,21,22,23], it remains unclear if this non-disease-relevant trend can be linked to the identified leaky c.544+5G>C *SHOX* splice-site variant, due to the limited sample size within this single pedigree and the high number of genes contributing to human growth. *SHOX* is known as a major growth gene that controls bone maturation, chondrocyte differentiation and proliferation, cellular growth arrest, and apoptosis by transcriptional regulation of its direct target genes *FGFR3*, *NPPB*, and *CTGF* [2,24,25,26,27]. In addition, SHOX promotes linear growth at the growth plate via direct interaction with SOX5, SOX6, and SOX9 [27,28]. Therefore, it is conceivable that heterozygous hypomorphic *SHOX* variants—such as the leaky c.544+5G>C *SHOX* splice-site variant—might contribute to population variability in human body proportions and height. Further studies in larger cohorts are needed to investigate the influence of hypomorphic *SHOX* variants to human growth and body proportions.

**Figure 3:**
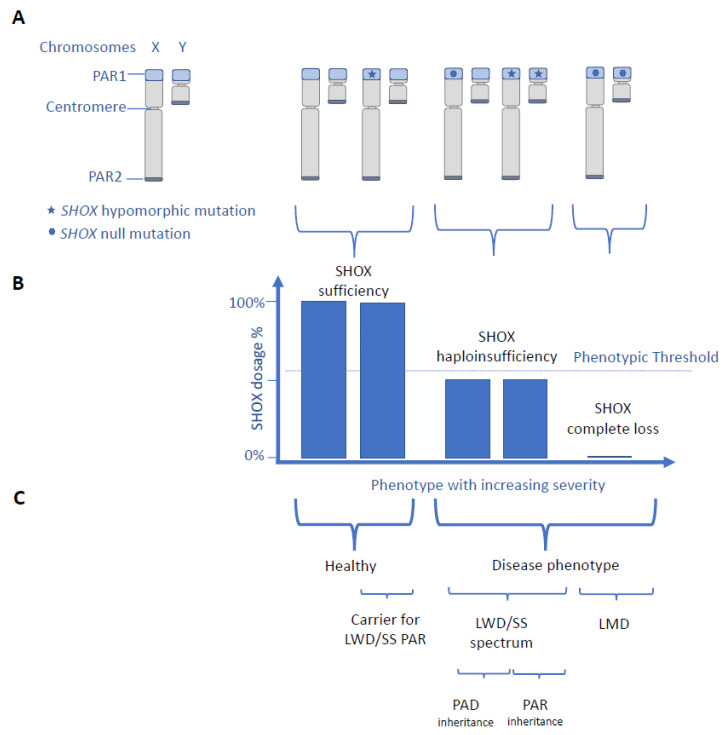
Scheme of human phenotypes in relation to *SHOX* genotypes and SHOX expression. (**A**) *SHOX* is located within the pseudo-autosomal region 1 (PAR1). Genes within PAR1 and PAR2 on the tips of both sex chromosomes are inherited such as autosomal genes. (**A**–**C**) Scheme of reported *SHOX* genotypes (**A**) in relation to SHOX expression (**B**) and human phenotypes (**C**). There is a SHOX-dosage-phenotype correlation with SHOX haploinsufficiency resulting in LWD/SS spectrum with pseudo-autosomal dominant (PAD) inheritance and complete loss of SHOX resulting in the severe LMD phenotype (**B**,**C**). In contrast *SHOX*-geno-phenotype, correlation is variable as biallelic *SHOX* variants can cause both, the LWD phenotype with pseudo-autosomal recessive inheritance (PAR) in case of biallelic hypomorphic *SHOX* variants inherited from healthy parents and the severe LMD phenotype in case of biallelic null variants inherited from clinically LWD-affected parents. Individuals without a SHOX disease phenotype might be carriers for pseudo-autosomal recessive LWD. In the presented model, the remaining SHOX transcript is assumed to represent the level of SHOX protein.

So far, the inheritance of LWD was considered a pseudo-autosomal dominant trait [3,4]. Dominant is defined as a genetic condition that manifests in the heterozygous state, irrespective of the clinical features in the homozygote. The term recessive inheritance pattern is used when a heterozygous variant is asymptomatic and if a disease phenotype arises only with pathogenic variants on both copies of a gene [29,30]. Our reporting pseudo-autosomal recessive inheritance of LWD with healthy heterozygotes is unprecedented as we demonstrate the underlying pathogenicity of a novel intronic *SHOX* variant, exclude the presence of additional *SHOX* variants, and exclude the concomitant presence of another chondrodysplasia in the index patient by WES. We are aware of a single previous report of supposedly pseudo-autosomal recessive LWD in a sib-pair, attributed to a homozygous 119-kb deletion downstream of the SHOX gene; no functional testing nor WES to exclude other reasons for short stature was applied [31]. Aggregate data from these two pedigrees ([31] and this study) show that biallelic *SHOX* variants are a rare cause of LWD and data from this study highlight that the dosage of SHOX determines the clinical phenotype rather than the Mendelian inheritance pattern of *SHOX* variants (Figure 3). This situation was reported for other human disease genes involving transcription factors such as *SALL1* causing Towns–Brocks syndrome (TBS) or Central-Nervous-System-TBS (MIM 107480) [32] and *TBX6* causing TBX6-associated congenital scoliosis or a spectrum of vertebral anomalies and Congenital Anomalies of Kidney and Urinary Tract (CAKUT) (MIM 122600) [33,34]. This supports the theory that the mutation type and strength and the gene dosage are fundamental in determining the rare disease traits rather than the classification of “recessive” or “dominant genes” [34,35]. Linking *SHOX* with pseudo-autosomal dominant and recessive patterns of inheritance is in accordance with the fact that a substantial number of genes traditionally associated with either recessive or dominant diseases are now linked to both inheritance patterns, based on functionally different pathogenic variants [30]. We therefore recommend considering both, the common pseudo-autosomal dominant and the rare pseudo-autosomal recessive inheritance in LWD, when counseling families and for variant interpretation in molecular *SHOX* testing. Importantly, the clinical expression in the four patients with pseudo-autosomal recessive LWD does not differ from LWD with pseudo-autosomal dominant inheritance considering the degree of short stature and skeletal involvement. This highlights the importance of a detailed family investigation and of functional testing of VUS in *SHOX,* to accurately distinguish between pseudo-autosomal dominant and recessive LWD.

It is well known that penetrance of SHOX haploinsufficiency is high while its clinical expression is highly variable and even among affected family members carrying the same familial *SHOX* variant [3,6,36]. Female sex and *CYP26C1* variants are known aggravating modifier for disease severity [3,37,38] but so far, no genotype–phenotype correlation has been reported for heterozygous pathogenic *SHOX* variants within the coding region [39] or within regulatory elements outside of the coding region [40,41]. Alternative splicing and NMD were hypothesized to contribute to time and tissue specific regulation of SHOX expression in human development [42]. Data from this study indicate that hypomorphic *SHOX* variants resulting in residual amounts of SHOX transcripts by NMD can attenuate the SHOX phenotype from LMD to LWD phenotype in case of biallelic hypomorphic *SHOX* variants and from SS/LWD spectrum to healthy individuals who are carriers for pseudo-autosomal recessive LWD. We speculate that hypomorphic *SHOX* variants might represent modifiers of disease severity (Figure 3). Further studies are needed to elucidate additional pathomechanisms contributing to phenotypic variability in SHOX deficiency and to further characterize the clinical spectrum of pseudo-autosomal recessive SHOX deficiency disorders.

In summary, this study extends the molecular and inheritance spectrum of SHOX deficiency disorder and highlights the importance of functional testing of *SHOX* variants of unknown significance in order to allow appropriate counseling and precision medicine for each family individual.

## Data Availability

The stated conclusions are based on the presented data of this study.

## Supplementary Materials

The following supporting information can be downloaded at: https://www.mdpi.com/article/10.3390/genes14040877/s1, Figure S1: Results of diagnostic testing for deletions and duplications of *SHOX* exons and regulatory elements.; Figure S2: The p.Val183Glyfs*31 *SHOX* variant results in premature stop codon.
