# Supplementary material for: Leri–Weill Dyschondrosteosis Caused by a Leaky Homozygous SHOX Splice-Site Variant
Source: Genes (Basel). 2023 Apr 7;14(4):877. doi: 10.3390/genes14040877 (PMC10138022; doi:10.3390/genes14040877)
Supplement: Supplementary file 1 [file genes-14-00877-s001.zip › genes-2282589-supplementary.pdf]

Supplemental File

1. SUPPLEMENTAL FIGURES

1.1. Supplemental Figure S1

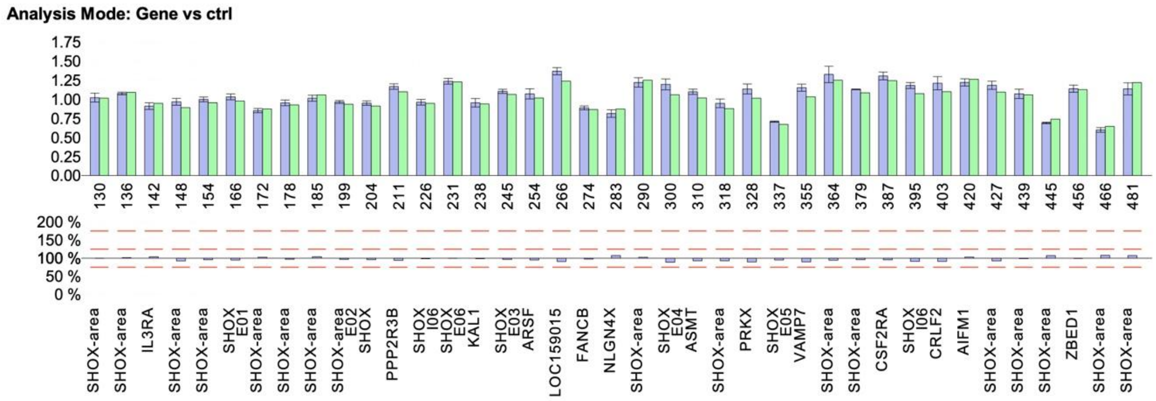

Supplemental Figure S1. Results of diagnostic testing for deletions and duplications of *SHOX* exons and regulatory elements. MLPA analysis using the commercial SALSA MLPA kit P018-G1 *SHOX* probemix (MRC-Holland) shows normal results in patient P1 (green boxes) when compared with controls (blue boxes).

1.2. Supplemental Figure S2

(

GRCH38

NC_000024.10(NM_000451.4):c.544+2_545dup ,

NC_000024.10:g.640880_640999dup ,

GRCH37

NC_000024.9(NM_000451.4):c.544+2_545dup ,

NC_000024.9:g.551615_551734dup ,

AFFECTED PROTEIN DESCRIPTION

NM_000451.4(NP_000442.1):p.(Val183_Leu292delinsGlyCysArgAspTrpGlyAspLeuLysLeu ,
GlyAspProAlaProGlyGlyMetGlySerThrArgCysTrpLeuHisProGlyProProHis)

AFFECTED PROTEIN REFERENCE SEQUENCE

MEELTAFVSKSFDQKSKDGNGGGGGGGGKKDSITYREVLESGLARSRELGTSDSSLQDITEGGGHCPVHLFKDHVDNDKEKLK
EFGTARVAEGIYECKEKREDVKSEDEDGQTKLKQRRSRTNFTLEQLNELERLFDETHYPDAFMREELSQRLGLSEARVQVWFQ
NRRAKCRKQENQMHKG**VILGTANHLDACRVAPYVNMGALRMPFQQVQAQLQLEGVAHAHPHLHPHLAAHAPYLMFPPPPFGLP**
IASLAESASAAAVVAAAAKSNSKNSSIADLRLKARKHAEALGL*

AFFECTED PROTEIN PREDICTED SEQUENCE

MEELTAFVSKSFDQKSKDGNGGGGGGGGKKDSITYREVLESGLARSRELGTSDSSLQDITEGGGHCPVHLFKDHVDNDKEKLK
EFGTARVAEGIYECKEKREDVKSEDEDGQTKLKQRRSRTNFTLEQLNELERLFDETHYPDAFMREELSQRLGLSEARVQVWFQ
NRRAKCRKQENQMHKG**GCRDWGDLKLGDPAPGGMGSTRCWLHPGPPH***

Supplemental Figure S2. The p.Val183Glyfs*31 *SHOX* variant results in premature stop codon. Using Mutalyzer (https://mutalyzer.nl/) the chromosomal position and the scoring of the intron 3 *SHOX* retention variant are shown. The wild-type (affected protein predicted sequence) is shown in relation to aberrant protein sequence (affected protein predicted sequence).

Supplemental results.

The WES pipeline identified 37581 variants following alignment to the GRCh37 reference sequence. Filtering for rare allele frequencies, and with HPO terms and as a Gene panel analysis revealed no pathogenic or likely pathogenic variants apart from the SHOX variant. Seven heterozygous variants of unknow significance were found in genes associated with syndromic skeletal disorders and dismissed because of lack of phenotypic overlap, and because they were present in heterozygous state where the known syndromic disorders were caused by biallelic variants.
